# Editorial: Emerging and Re-emerging Vector-borne and Zoonotic Diseases

**DOI:** 10.3389/fmed.2021.714630

**Published:** 2021-08-05

**Authors:** Alfonso J. Rodriguez-Morales, Jaime A. Cardona-Ospina, Matthew H. Collins

**Affiliations:** ^1^Grupo de Investigación Biomedicina, Faculty of Medicine, Fundacion Universitaria Autonoma de las Americas, Pereira, Colombia; ^2^Emerging Infectious Diseases and Tropical Medicine Research Group, Instituto para la Investigación en Ciencias Biomédicas - Sci-Help, Pereira, Colombia; ^3^School of Medicine, Universidad Privada Franz Tamayo (UNIFRANZ), Cochabamba, Bolivia; ^4^Faculty of Health Sciences, Universidad Científica del Sur, Lima, Peru; ^5^Division of Infectious Diseases, Department of Medicine, Emory University School of Medicine, Atlanta, GA, United States

**Keywords:** vector-borne diseases, zoonotic diseases, emerging, tropical diseases, emerging infectious diseases, global health, spillover, pandemic

The Colombian Nobel laureate Gabriel Garcia Marquez stated in his last will, “*la muerte no llega con la vejez, sino con el olvido*” (“death does not come with old age, but with oblivion”). Indeed, how many deaths due to tropical diseases can be avoided? How can investment in these neglected diseases significantly change the course of the disease? Even in a macro vision, how could the socioeconomic condition of those affected by these diseases be changed to avoid transmission, morbidity, and mortality? We must rescue tropical and emerging global diseases from oblivion, and the rescue begins with us.

Despite significant advances in diagnostic tools, sequencing technologies (1–5), new drugs, and vaccine development using precision medicine (6–9), pharmacogenomics (10–12), computational and *in silico* models (13–16), and artificial intelligence (17–20); the benefits of these accomplishments have not been fully realized in the field of emerging and re-emerging vector-borne and zoonotic diseases (21). Emerging vector-borne and zoonotic diseases are a growing threat to global health and have caused hundreds of billions of US dollars of economic damage in the past 20 years (22). Together, these infections are responsible for a substantial disease burden, with endemic and enzootic zoonoses, and metaxenic diseases causing about a billion cases of illness in people and millions of deaths every year (22). Moreover, old foes with us for hundreds [like Chagas disease (23–25)] or thousands [like leprosy or Hansen's disease (26–28)] of years are yet to be eliminated or controlled in many countries. This is the tragedy of neglected tropical diseases. Disinterest and disincentive are monstrous impediments to the progress that could be made by governments, major pharmaceutical companies, and other actors in the development of new drugs, research initiatives, diagnostics, and vaccines for these diseases.

The rescue from oblivion is accomplished in multiple ways, including increasing visibility; generating, disseminating, and implementing new knowledge and evidence; elaborating strategies and tools for the benefit of communities and patients, and pursuing research in a more integrated and comprehensive form, but also looking for ways to translate that research into policies. After millennia, standing in the dawn of the XXI century, we are at a crossroads. We can reinvigorate the global commitment to confront these problems; otherwise, several of these conditions will persist, taking millions of lives, causing substantial disability, and increasing poverty in already impoverished populations.

This collection of articles endeavors to identify unique and essential challenges and opportunities for improving the management of vector-borne and zoonotic diseases. As clinician-investigators, we favor a future that is characterized by improved health metrics globally and a human population that seeks to be a good steward of the planet on which it depends. Of course, it is easy to pontificate about what must be done and how grand the scale should be. However, viable solutions will accept the arduous tasks that buttress their pursuit. Our world is one of limited capital (political, human, financial). Thus, the field of vector-borne and zoonotic diseases needs to include advocates, educators, and communicators to ensure that populations have awareness and understanding of what is at stake for their future and political leaders are held accountable to serve the best interest of their people through policy and resource allocation (29–31). Here, we outline vital ideas that will shape the future of the vector-borne and zoonotic diseases field. We draw on One Health and Planetary Health models—essentially recognizing the interdependence of human health, animal health, and environmental health; ideas endorsed by major international organizations such as WHO (32)—and include lessons learned from the contributors to this collection of articles.

The diagnostics underlying surveillance are a cornerstone for the public health management of infectious diseases, providing a means to monitor and model transmission and evaluate the impact of prevention and control activities (33). However, existing systems vary considerably in the organization, investment, and linkage to action or resource mobilization. Surveillance networks often focus on a specific pathogen but may also be centered on the syndromic presentation of illnesses such as acute respiratory illness or hemorrhagic fever. While serologic approaches remain important, molecular surveillance is the nexus of pathogen discovery and monitoring. Global surveillance must be strengthened at all levels. Bolstering existing vertical systems at local and national levels has advantages. These include investment in training and deployment of skilled field epidemiologists and maintenance of information systems for collecting, analyzing, and reporting data. Complimentary efforts by international entities such as the WHO to standardize practices and definitions help coordinate response efforts. Given the high volume of (pre-2020) international travel, unique surveillance networks that precisely monitor travel-related infections offer exciting opportunities to assess the spread of infectious diseases across borders (34–36). Of course, case definitions must be in place and diagnostic tools must be rapidly developed and deployed for novel pathogens to enable accurate surveillance. Making surveillance information robust and readily accessible accelerates research to understand and respond to existing and novel pathogens while informing public health decisions.

Treatment and prevention of infections are critical. Vaccination, which is not covered further here due to space limitations, is among the most celebrated achievements in medicine, and recent efforts in the Zika and COVID-19 pandemics have set an incredibly high bar for the rapidity with which safe and effective vaccine candidates can be developed (37). There are no licensed antivirals and a dearth of promising candidates for emerging diseases such as SARS, MERS, Ebola and Zika (38). The trend has been that intense research and publications follow on the heels of news of a new epidemic of potential global concern. However, interest and investment in these activities quickly subside. A greater armamentarium of treatments could have enormous benefits for combatting future vector-borne and zoonotic diseases, which is precisely the goal of innovative initiatives such as READDI (Rapidly Emerging Antiviral Drug Discovery Initiative) (38). Completing phase 1 trials of antivirals does not require a pre-known indication. We could start at phase 2, quickly screen *in vitro*, and test *in vivo* in parallel.

This Research Topic by *Frontiers in Medicine* and *Frontiers in Public Health* brings together a diversity of articles that focus on different pathogens, representing different points on the translational research spectrum, anchored in different disciplines. The goal was to provide a succinct sample of the vector-borne and zoonotic diseases affecting populations worldwide and some of the scientific methods involved in a public health response. The collection was finalized in 2019; but, the events of 2020 cannot be ignored. The COVID-19 pandemic is nothing less than a calamity. However, the emergence of SARS-CoV-2 is neither unique nor surprising; it is part of a pattern and process, and the palpable consequence of the close correlation of human, environmental, and animal health. Details of emergence are difficult to predict, but the reality that our global ecosystem holds countless potential pathogens, particularly RNA viruses, that could “spillover” under certain conditions and propagate among humans is well-known. The possible emergence of other CoV epidemics was predicted in the prescient work by Menachery et al., several years before the current pandemic (39). The COVID-19 pandemic has been difficult and tragic, but it also reveals the need for dedicated attention to comprehensive management plans for vector-borne and zoonotic diseases that could prevent future catastrophes.

This Research Topic has exceeded expectations in scope and content. The original article on dengue is an exciting paper, combining findings and experiences from two endemic countries in Latin America (Ecuador) and Asia (Thailand) Anderson et al. The Zika virus papers show the importance of surveillance and the broad spectrum of congenital disorders associated with this arbovirus, as is the case of arthrogryposis Contreras-Capetillo et al. and Lim et al. In addition to the epidemiological and clinical aspects of arboviruses, basic immunological aspects are also crucial in understanding these conditions, as shown in the article about T cell responses to Japanese encephalitis Pushpakumara et al. After the 2014 epidemics, Ebola persisted in countries such as the Democratic Republic of Congo, representing a significant burden of morbidity and mortality, as discussed in the article by Grimes et al. Myiasis, although forgotten by many, is still causing problems in different populations, including newborns, as discussed in the article by Ruiz-Zapata et al. The role of dogs in multiple zoonotic diseases still needs to be addressed regarding multiple pathogens, including *Echinococcus granulosus*, as presented by Khan et al., in their article. Coinfections represent a challenge in the context of tropical diseases related to other tropical diseases, but also other infectious diseases; this interesting aspect in the context of HIV is presented in the diagnosis of visceral leishmaniasis da Silva et al. In its vision of ecoepidemiology, the article of Krystosik on the role of solid waste in breeding sites, burrows, and food for vectors and urban zoonotic reservoirs is fascinating Krystosik et al.

It seems clear that zoonotic and emerging infectious diseases must be confronted via a multifaceted approach, which includes integrating across disciplines (veterinary medicine, vector biology, immunology, epidemiology, among others) as well as across biological scales (molecules → pathogens → ecosystems). Among the best existing frameworks to improve integration in our concepts, health offers new collaborations and actions. We will undoubtedly be given additional opportunities to react to small outbreaks or new pandemic threats (32, 40–43). However, success in this field will be marked by an increasingly proactive and preemptive mode of operation—we need to know what is coming, have adequate tools and therapeutics poised for application and adaptation, and identify measures to prevent the emergence of novel pathogens (rather than the spread of emerging diseases)—particularly as a counter to myopic or destructive human activity, as these are a detrimental driver for the health of our species.

Many are hard at work, but more effort is required and the challenges are great. We want a world with equity, less disease, and more health, especially in those areas where the impact of these conditions has truncated the lives of millions. We hope readers will benefit from the insights of the experiences and findings in this Research Topic whilst also being motivated to take action in pulling these diseases, and those who suffer from them, out of oblivion.

## Author Contributions

All authors contributed to manuscript conception and design, literature review, manuscript preparation, critical review, and contributed to the article and approved the submitted version.

## Conflict of Interest

The authors declare that the research was conducted in the absence of any commercial or financial relationships that could be construed as a potential conflict of interest.

## Publisher's Note

All claims expressed in this article are solely those of the authors and do not necessarily represent those of their affiliated organizations, or those of the publisher, the editors and the reviewers. Any product that may be evaluated in this article, or claim that may be made by its manufacturer, is not guaranteed or endorsed by the publisher.
